# Cost-effectiveness of the SLIMMER diabetes prevention intervention in Dutch primary health care: economic evaluation from a randomised controlled trial

**DOI:** 10.1186/s12913-019-4529-8

**Published:** 2019-11-11

**Authors:** Geerke Duijzer, Andrea J. Bukman, Aafke Meints-Groenveld, Annemien Haveman-Nies, Sophia C. Jansen, Judith Heinrich, Gerrit J. Hiddink, Edith J. M. Feskens, G. Ardine de Wit

**Affiliations:** 1 Wageningen University, Division of Human Nutrition; Academic Collaborative Centre AGORA, PO Box 17, 6700 AA, Wageningen, the Netherlands; 2 GGD Noord- en Oost-Gelderland (Community Health Service), PO Box 3, 7200 AA, Warnsveld, the Netherlands; 3 Strategic Communication, Sub-department Communication, Philosophy and Technology: Centre for Integrative Development, Social Sciences, Wageningen University, PO Box 8130, 6700 EW, Wageningen, the Netherlands; 4 Julius Center for Health Sciences and Primary Care, University Medical Center Utrecht, Julius Centrum, PO Box 85500, STR 6.131, 3508 GA, Utrecht, the Netherlands; 5 Centre for Nutrition, Prevention and Healthcare, National Institute for Public Health and the Environment (RIVM), PO Box 1, 3720 BA, Bilthoven, the Netherlands; 6 Wageningen University, Consumption and Healthy Lifestyles, PO Box 8130, 6700 EW, Wageningen, the Netherlands

**Keywords:** Cost-effectiveness, Diabetes, Prevention, Lifestyle intervention, Economic evaluation

## Abstract

**Background:**

Although evidence is accumulating that lifestyle modification may be cost-effective in patients with prediabetes, information is limited on the cost-effectiveness of interventions implemented in public health and primary health care settings. Evidence from well-conducted pragmatic trials is needed to gain insight into the realistic cost-effectiveness of diabetes prevention interventions in real-world settings. The aim of this study is to assess the cost-effectiveness of the SLIMMER lifestyle intervention targeted at patients at high risk of developing type 2 diabetes compared with usual health care in a primary care setting in the Netherlands.

**Methods:**

Three hundred and sixteen high-risk subjects were randomly assigned to the SLIMMER lifestyle intervention or to usual health care. Costs and outcome assessments were performed at the end of the intervention (12 months) and six months thereafter (18 months). Costs were assessed from a societal perspective. Patients completed questionnaires to assess health care utilisation, participant out-of-pocket costs, and productivity losses. Quality Adjusted Life Years (QALY) were calculated based on the SF-36 questionnaire. Cost-effectiveness planes and acceptability curves were generated using bootstrap analyses.

**Results:**

The cost-effectiveness analysis showed that the incremental costs of the SLIMMER lifestyle intervention were €547 and that the incremental effect was 0.02 QALY, resulting in an incremental cost-effectiveness ratio (ICER) of €28,094/QALY. When cost-effectiveness was calculated from a health care perspective, the ICER decreased to €13,605/QALY, with a moderate probability of being cost-effective (56% at a willingness to pay, WTP, of €20,000/QALY and 81% at a WTP of €80,000/QALY).

**Conclusions:**

The SLIMMER lifestyle intervention to prevent type 2 diabetes had a low to moderate probability of being cost-effective, depending on the perspective taken.

**Trial registration:**

The SLIMMER study is retrospectively registered with ClinicalTrials.gov (Identifier NCT02094911) since March 19, 2014.

## Background

Nowadays, diabetes is recognised as a major public health problem as it leads to a high disease and economic burden. In 2013 alone, diabetes accounted for 5.1 million deaths and a global health expenditure of USD 548 billion (11% of total health expenditure) [1]. Diabetes is associated with unhealthy lifestyle characteristics, including obesity, poor diet, and physical inactivity [1]. Although evidence is accumulating that lifestyle modification may be cost-effective in patients with prediabetes, information is limited on the cost-effectiveness of interventions implemented in public health and primary health care settings [2]. Evidence from well-conducted pragmatic trials is needed to gain insight into the realistic cost-effectiveness of diabetes prevention interventions in real-world settings. The Dutch SLIM intervention, which led to a 47% diabetes risk reduction amongst study participants [3], has proven to be cost-effective [4]. The SLIM intervention was subsequently translated from the experimental setting into a real-world intervention, called SLIMMER [5–7]. The aim of the current study is to assess the cost-effectiveness of the SLIMMER lifestyle intervention compared with usual health care in a primary care setting. We recently reported the effects of the SLIMMER intervention, including improvements in anthropometry, glucose metabolism, dietary intake, physical activity, and quality of life. These improvements were more significant amongst the intervention group than in the control group, both at 12 and at 18 months [8]. Here, we report on the cost-effectiveness analysis conducted alongside this pragmatic randomised trial.

## Methods

### Study design

The design of the SLIMMER study has been published in detail elsewhere [6]. In short, the SLIMMER study is a randomised controlled trial carried out in Dutch primary health care between 2011 and 2014. We performed an economic evaluation from a societal perspective.

### Study population and setting

Twenty-five general practices (general practitioners, GPs, and practice nurses) recruited patients aged between 40 and 70 years at increased risk of diabetes, defined as having impaired fasting glucose (IFG; 6.1–6.9 mmol/l [9]) or an elevated/high risk of type 2 diabetes (a Diabetes Risk Test score of ≥7 points [10]). The study was conducted in the Dutch cities Apeldoorn and Doetinchem. The SLIMMER intervention was implemented in primary health care, involving GPs and their practice nurses, dieticians, physiotherapists, and local sports clubs. The existing structure of GPs, having natural referral lines with dieticians and physiotherapists, was used for implementation of the SLIMMER intervention. The study protocol was approved by the Wageningen University Medical Ethics Committee, and all subjects gave their written informed consent before the start of the study. The SLIMMER study is registered with ClinicalTrials.gov (Identifier NCT02094911).

### Randomisation procedure

After baseline measurement, 316 participants were randomly allocated to the intervention or to the control group, using block randomisation at GP level and stratification for sex. Spouses were allocated to the same group to avoid contamination. An independent dietician from the Division of Human Nutrition, Wageningen University, performed the randomisation.

### Control group

At the time of the study, all Dutch patients with impaired fasting glucose or an elevated/high risk of type 2 diabetes received usual health care from their GPs and/or practice nurses. This was conform the standard for prevention of type 2 diabetes in primary health care, developed by the Dutch College of General Practitioners [12]. Therefore, the control group received this usual health care, consisting of yearly monitoring of blood glucose. Furthermore, at baseline, the control subjects received written information on the beneficial effects of a healthy diet and increased physical activity.

### Lifestyle intervention

Besides usual health care provided by GPs and/or practice nurses, the intervention group received the SLIMMER lifestyle intervention. This intervention resembled the SLIM intervention [3] and consisted of a dietary and physical activity intervention, including case management and a maintenance programme. The SLIMMER intervention conformed regular functioning and professional performance of Dutch GPs, practice nurses, dieticians, and physiotherapists. Minimal training and a detailed manual were provided during a two-hour SLIMMER kick-off training for health care professionals. The dietary intervention consisted of five to eight individual consultations and one group session with a dietician during 10 months. Tailored dietary advice was given on a sustainable healthy dietary pattern and during the group session participants shared experiences, motivated each other, and discussed the topic of label reading. The physical activity intervention was delivered by physiotherapists as weekly group-based training sessions for 10 months and consisted of both aerobic and resistance exercise. Physical activity training groups were formed after randomisation, based on day and time preferences of participants and availability of physiotherapists. Groups with a minimum number of four participants then started the intervention programme. Furthermore, case management was performed by practice nurses and consisted of keeping in contact with both health care professionals and intervention participants throughout the intervention period, to detect and solve problems, and to motivate and support participants. In addition to this core programme, a maintenance programme was delivered during the last phase of the intervention period and continued up to three months after the end of the intervention. This maintenance programme comprised of sports clinics at local sports clubs and concluding meetings with the dietician and physiotherapist during the core programme of 10 months, and a return session with the dietician, physiotherapist, and the physical activity group three months after the end of the intervention [11].

### Data collection and outcomes

#### Measurements

Participants visited the research centre for measurements at baseline, directly after the intervention (12 months), and at 18 months. Participants completed questionnaires at each visit to assess health care utilisation, participant out-of-pocket costs, productivity losses, and quality of life. The present cost-effectiveness analysis (CEA) includes effects and costs over the total 18-month study period.

#### Volumes of resources used

Data on volumes of health care utilisation (general practitioner, dietician, physiotherapist, consultations at outpatient clinic, and hospitalisation), use of medication, and participant out-of-pocket costs (sports club memberships and sports equipment) were obtained from participant questionnaires that were collected intermittently (at baseline, 12 and 18 months) with a 3-month recall period. Productivity losses (related to both absence from work, absenteeism, and less productivity while working, presenteeism) were measured using the Short Form Health and Labour Questionnaire (SF-HLQ) [13].

#### Cost prices

We used 2012 price levels, since the intervention was delivered mainly in 2012, and indexed prices when necessary using the consumer price indices from Statistics Netherlands [14]. Discounting was not applied because of the short timeframe of 18 months. A detailed description of cost prices is given in Additional file 1.

#### Intervention costs

Bottom-up micro-costing analysis was used to estimate intervention costs as this method is advised in health economic guidelines [15]. Intervention costs were estimated in a realistic way by dividing total costs over those who completed the programme. Selection and recruitment of participants cost €37 per participant. Intervention materials were valued using charges paid. Training of GPs and practice nurses and supervision by a project coordinator cost €133 per participant. The volumes of individual and group dietary sessions, physical activity sessions, and the return session were collected by attendance registration. These volumes were multiplied by unit prices for each component of the lifestyle intervention. Cost prices per unit were retrieved from the Dutch guideline for costing analysis in health care [15, 16].

#### Utilities and quality adjusted life years (QALY)

The Short-Form Health Survey (SF-36) [17, 18] was used to assess quality of life at every visit to the research centre. Health utilities were determined by the SF-6D health state classification [19], a preference-based single index derived from the SF-36. QALYs were used as main outcome parameter for the economic evaluation and calculated by multiplying health utilities by the amount of time a participant spent in a particular health state. Transitions between health states were linearly interpolated.

### Statistical analyses

A sample size of 145 subjects per group was required to detect differences between groups in fasting insulin, assuming an alpha of 0.05, power of 80%, two-sided test, and an expected drop-out rate of 10%. Intention to treat analyses were performed. Missing cost and outcome data (16%) were imputed with multiple imputation techniques, using Fully Conditional Specification and Predictive Mean Matching procedures. The imputation model included age, sex, baseline health status, randomisation group, and available costs and outcomes at each measurement. Baseline characteristics were compared with an independent samples *t* test for normally distributed data, a Mann-Whitney test for non-normally distributed data, and a Pearson’s chi-squared test for categorical data.

#### Economic analyses

The incremental cost-effectiveness ratio (ICER) was calculated as the difference in costs divided by the difference in QALYs between the intervention and the control group using a bootstrap analysis with 1000 simulations. From the bootstrap analysis, a cost-effectiveness plane was plotted, where each quadrant indicates whether the intervention is more or less effective and more or less expensive than usual health care. Furthermore, cost-effectiveness acceptability curves (CEACs) were plotted to illustrate the uncertainty of cost-effectiveness estimates. The CEAC shows the probability that the SLIMMER intervention is cost-effective compared with usual health care, for a range of threshold values for willingness to pay (WTP) per additional QALY. In the Netherlands, threshold values of €20,000 to €80,000 per QALY are commonly used [20].

#### Sensitivity analyses

Sensitivity analyses were performed to assess cost-effectiveness using different input parameters. In the first sensitivity analysis, cost-effectiveness was calculated from a health care perspective, taking into account only intervention costs and direct health care costs. The second sensitivity analysis was restricted to participants with complete cost and effect data, that is, complete cases. In the third sensitivity analysis, intervention costs were reduced. If the SLIMMER intervention would be implemented regularly in health care, the project coordinator would be redundant, therefore these costs were excluded.

## Results

**Fig. 1:**
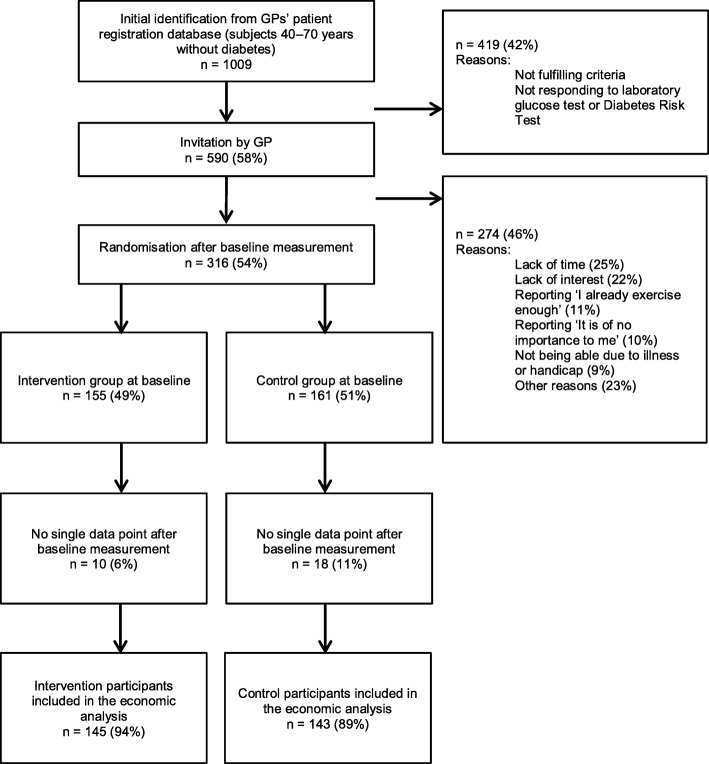
Flow diagram of the SLIMMER randomised controlled trial, for cost-effectiveness analyses

For the economic analysis, data for 288 (91%) SLIMMER study participants were available (Fig. 1). Twenty-eight participants were excluded because they did not complete a single questionnaire nor were other measurements available. As shown in Table 1, baseline characteristics were similar between the intervention and the control group.

**Table 1:** Baseline characteristics of the SLIMMER study participants included in the cost-effectiveness analyses^a^

	Intervention (*n* = 145)	Control (*n* = 143)
Sex (*n* female, %)	67 (46%)	71 (50%)
Age (years)	60.9 ± 6.0	61.1 ± 6.5
Education level^b^ (*n*, %)		
Low	77 (53%)	76 (53%)
Middle	40 (28%)	28 (20%)
High	28 (19%)	39 (27%)
Cultural background (*n*, %)		
Dutch	128 (88%)	129 (90%)
Western non-Dutch	13 (9%)	11 (8%)
Non-western non-Dutch	4 (3%)	3 (2%)
Family history of diabetes (*n*, %)		
No	46 (32%)	61 (43%)
First degree	70 (48%)	65 (45%)
Second degree	29 (20%)	17 (12%)
Paid job^c^ (*n*, %)	67 (46%)	68 (48%)
Smoking (*n*, %)	22 (15%)	27 (19%)
BMI (kg/m^2^)^d^	30.3 ± 4.6	29.9 ± 4.8
Waist circumference (cm)^d^		
Male	109.1 ± 12.2	107.8 ± 10.1
Female	101.3 ± 12.9	99.9 ± 12.6
Fasting glucose (mmol/l)	6.6 ± 0.6	6.5 ± 0.6
2-h glucose (mmol/l)^d^	8.2 ± 2.8	8.0 ± 2.5
Fasting insulin (pmol/l)	89.6 ± 51.7	84.8 ± 52.2
SF-6D health state	0.79 ± 0.12	0.79 ± 0.10

^a^Data are mean ± SD, or *n* (%)

^b^Education level was based on the highest level of education completed and divided in three categories: low (no, primary or lower secondary school), middle (higher secondary school or intermediate vocational school), and high (higher professional education or university level)

^c^Paid job includes both full time and part time jobs

^d^INT *n* = 144, CON *n* = 143

### Costs

Table 2 shows costs of the intervention and the control subjects. Total costs of the intervention were €677 per participant. Costs for the intervention, participant out-of-pocket costs, and costs for absenteeism were higher in the intervention group than in the control group, whereas costs for hospitalisation, medication, and presenteeism were lower. The incremental cost difference between groups was €547.

**Table 2:** Mean (standard deviation) costs for intervention and control subjects

		Intervention (*n* = 145)	Control (*n* = 143)
	Unit costs (€)	Mean total costs^a^ € (SD)	Mean total costs^a^ € (SD)
*Intervention costs*			
Selection and recruitment by practice nurse	37.08 per participant	37 (0)	0 (0)
Materials	15.65 per participant	16 (0)	0 (0)
Project coordinator	133.02 per participant	133 (0)	0 (0)
Individual consultations with dietician	28.64 per hour	101 (18)	0 (0)
Group session with dietician	6.20 per session	4 (3)	0 (0)
Group-based training sessions with physiotherapist	8.06 per session	319 (161)	0 (0)
Sports clinics at local sports club	24.69 per sports clinic	60 (47)	0 (0)
Return session with dietician and physiotherapist	8.92 per session	6 (4)	0 (0)
*Subtotal*		*677 (194)*	*0 (0)*
*Direct health care costs*			
Consultations general practice	Additional file 1	118 (150)	190 (193)
Consultations dietician	28.64 per hour	2 (8)	9 (45)
Consultations physiotherapist	38.18 per hour	111 (319)	94 (246)
Consultations health care specialist	76.38 per visit	291 (494)	272 (420)
Hospital days	484.72 per day	426 (1758)	637 (4467)
Medication	Individualised	369 (458)	526 (659)
*Subtotal*		*1317 (2138)*	*1728 (4953)*
*Direct non-health care costs*			
Sports club membership	Individualised	233 (419)	224 (370)
Sports equipment	Individualised	151 (339)	112 (425)
*Subtotal*		*384 (595)*	*336 (573)*
*Indirect non-health care costs*			
Absence from work	Individualised	1995 (8600)	1285 (6859)
Less productivity while working	Individualised	500 (2164)	975 (4390)
*Subtotal*		*2495 (9183)*	*2261 (8725)*
Total costs (€)			
Societal perspective	–	4872 (10,281)	4325 (10,612)
Health care perspective	–	1993 (2144)	1728 (4953)

^a^Total costs represent costs over the total 18-month study period

### QALYs

Participants’ health status at baseline was comparable between the intervention and the control group, whereas it was higher in the intervention group than in the control group after 12 and 18 months, albeit non-significantly. Total QALY over the 18-month study period was 0.02 (− 0.01; 0.05) higher in the intervention group than in the control group (Table 3).

**Table 3:** Mean health-related quality of life at the end of the intervention (12 months) and at 18-month follow-up and the QALYs for the intervention and the control group

	Intervention (*n* = 145)	Control (*n* = 143)	Mean difference
	Mean (SD)	Mean (SD)	Mean (95% CI)
SF-6D health status			
Baseline	0.79 (0.12)	0.79 (0.10)	−0.001 (−0.03; 0.02)
12 months	0.81 (0.11)	0.79 (0.11)	0.02 (− 0.002; 0.05)
18 months	0.80 (0.13)	0.79 (0.12)	0.01 (−0.02; 0.04)
QALY total over 18 months	1.20 (0.15)	1.19 (0.14)	0.02 (−0.01; 0.05)

### Economic analyses

**Fig. 2:**
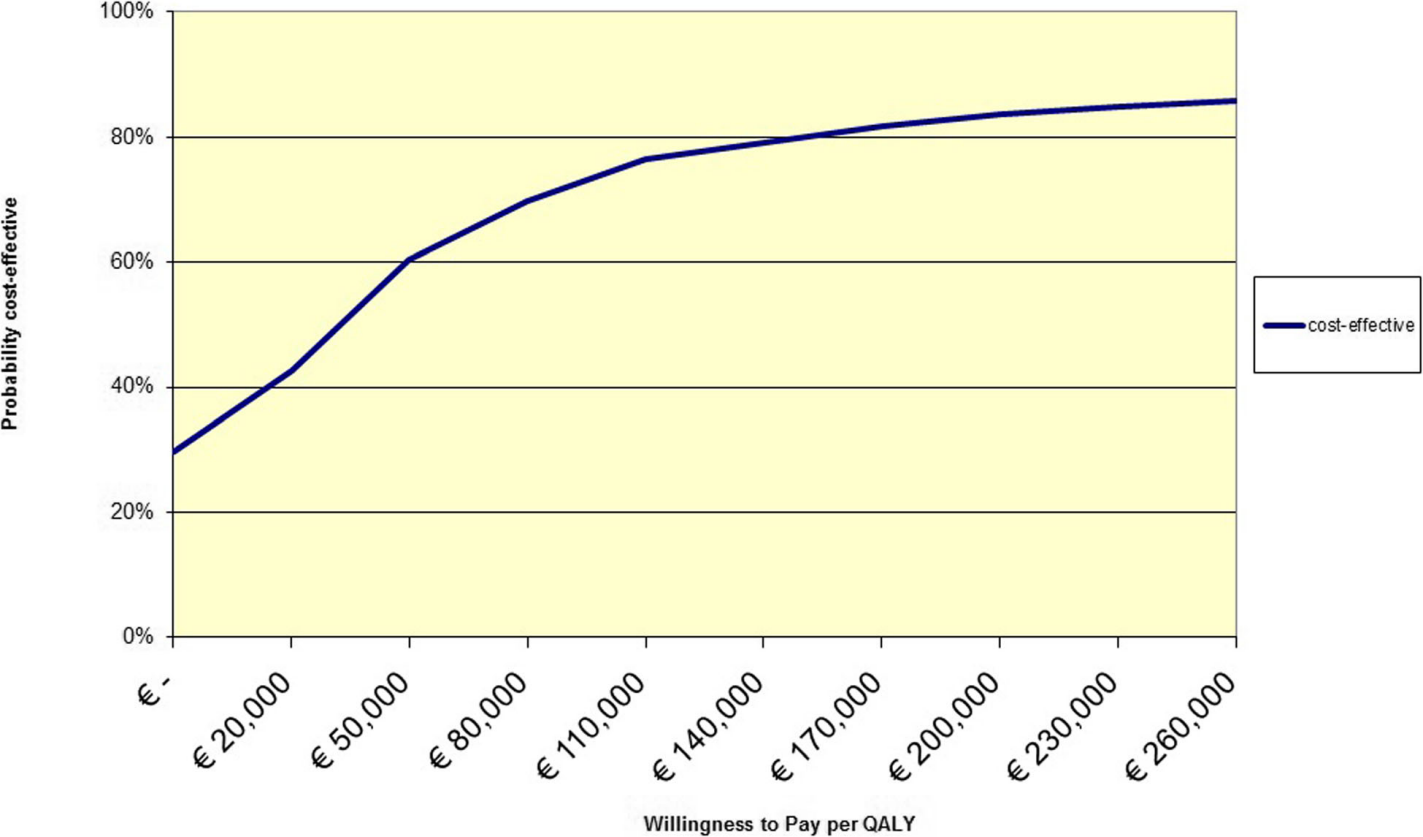
Cost-effectiveness acceptability curve of the SLIMMER intervention compared to usual health care, from a societal perspective

The higher costs and effects in the intervention group compared with the control group resulted in an ICER of €28,094/QALY (Table 4). From the bootstrap analysis, it appeared that most simulations showed higher costs for the intervention as well as small positive QALY differences between the intervention and the control group (Additional file 2). Figure 2 shows that, if society is willing to pay either €20,000 or €80,000 per additional QALY, the probability that the intervention will be cost-effective is 43 and 70%, respectively.

**Table 4:** Results of sensitivity analyses

	Sample size per group		Incremental effect	Incremental costs	ICER	Dominance	Probability cost-effective (WTP^a^ €20,000/QALY)	Probability cost-effective (WTP^a^ €80,000/QALY)
	Intervention	Control	QALY	€	€/QALY	%	%	%
Societal perspective	145	143	0.02	547	28.094	30	43	70
Health care perspective	145	143	0.02	265	13.605	26	56	81
Complete cases	123	119	0.02	600	24.586	32	48	75
Reduced intervention costs	145	143	0.02	414	21.266	34	47	73

^a^*WTP* Willingness to pay

### Sensitivity analyses

**Fig. 3:**
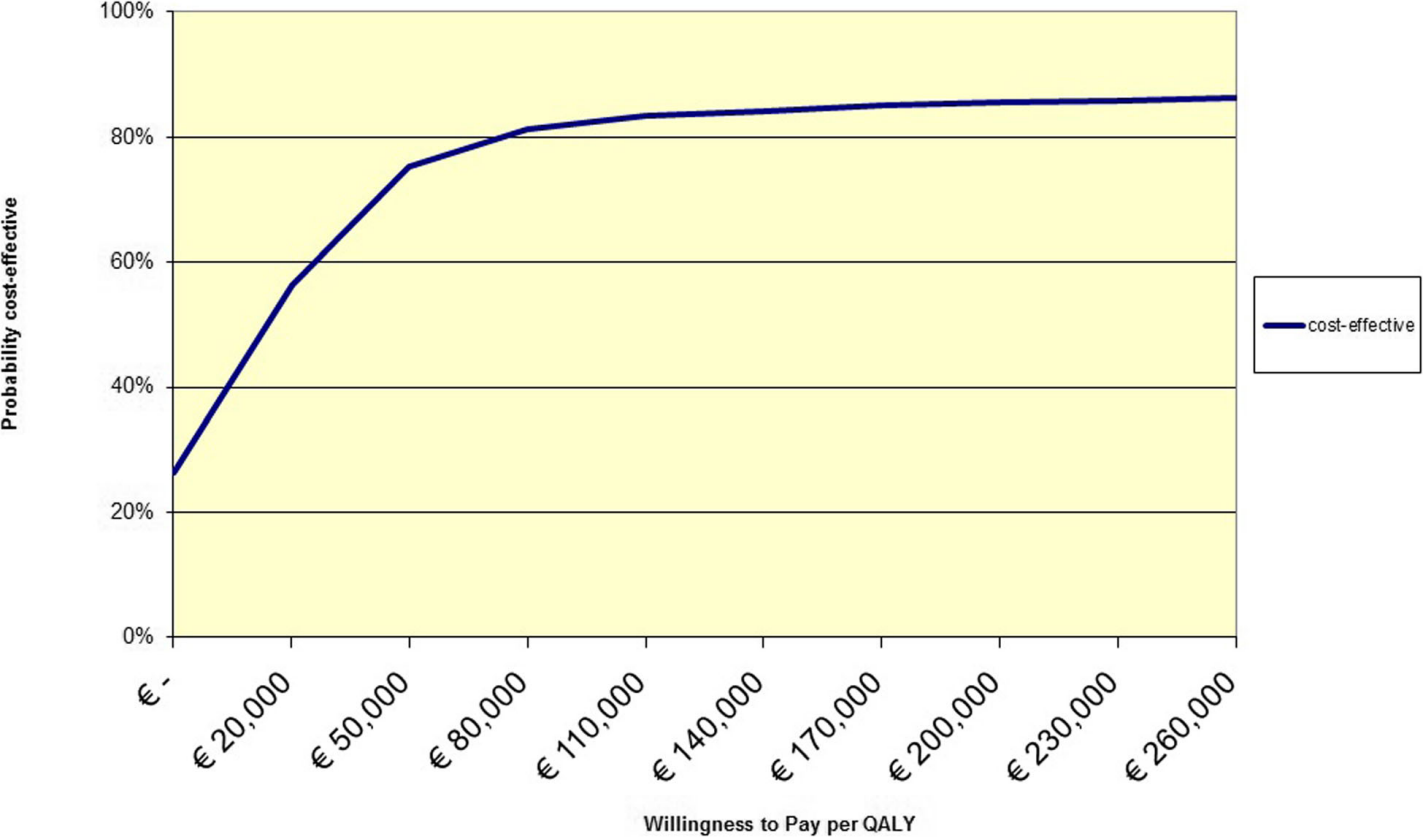
Cost-effectiveness acceptability curve of the SLIMMER intervention compared to usual health care, from a health care perspective

The sensitivity analyses for complete cases and reduced intervention costs revealed similar results as the base case analysis (Table 4). However, when cost-effectiveness was calculated from a health care perspective, the ICER decreased to €13,605/QALY (Table 4). The probability of the intervention being cost-effective was 56% at a WTP of €20,000/QALY and 81% at a WTP of €80,000/QALY (Fig. 3).

## Discussion

The current study showed that the SLIMMER intervention was both more costly and more effective than usual health care. As expected, the intervention group had a lower health care utilisation and reported less presenteeism than the usual care group. From a societal perspective, the ICER was €28,094/QALY, reflecting a relatively low probability of cost-effectiveness of 43% at a WTP of €20,000/QALY and a higher probability of 70% at a WTP of €80,000/QALY. From a health care perspective, the ICER was €13,605/QALY, with a moderate probability of being cost-effective (56% at a WTP of €20,000/QALY and 81% at a WTP of €80,000/QALY).

Nowadays, more and more insight into the cost-effectiveness of diabetes prevention programmes is becoming available. Recently, a systematic review found a median ICER for diet and physical activity programmes of $13,761/QALY (2013 price levels), from a health care perspective [2]; this is comparable to our ICER. Most of the studies included in that review were based on the US Diabetes Prevention Program (DPP) or the Finnish Diabetes Prevention Study (DPS), like our DPS-based SLIMMER study. However, a Dutch study that investigated the cost-effectiveness of a primary care lifestyle intervention for prevention of type 2 diabetes and cardiovascular disease showed that the intervention was cost-saving without being effective [21]. Another Dutch study, on the prevention of weight gain among employees, failed to reveal cost-effectiveness too [22]. The Dutch SLIM study which formed the basis of our intervention programme revealed an ICER of €3900–€5500 [4]. Results, however, are difficult to compare due to methodological differences, such as the lifetime horizon and modelling with SLIM, and the different years of cost.

The higher costs in the intervention group were due mainly to costs of the intervention programme. We should therefore consider possibilities to reduce these intervention costs, like appointing sports instructors instead of higher-salaried physiotherapists. The Greaves et al.’s review [23] showed that a wide range of providers can deliver effective interventions. Furthermore, the provision of group-based dietary consultations could be considered, as Li et al.’s review showed that group-based interventions were less costly and more cost-effective than individual-based interventions [2]. In addition, an even more individually tailored intervention approach could be used by referring participants earlier to regular sports clubs when they are ready to do so, rather than adhering strictly to the programme’s schedule. These adaptations were not taken into account in the sensitivity analysis because the impact of changes in the intervention on its effectiveness is currently unknown. Further research on this issue is necessary.

Costs for health care utilisation, mainly hospitalisation and medication use, and presenteeism were lower in the intervention group than in the control group. This was also found in the DPP study [24] and the Dutch Hoorn Prevention study [21]. The reduced direct health care costs indicate that the benefits of this intervention may be attractive for health insurance companies. Unexpectedly, costs for absenteeism were higher in the intervention group than in the control group. More detailed inspection of the causes of absenteeism revealed that these productivity losses were in general unrelated to physical fitness or diabetes, but for example to fever. Hence, the higher productivity costs in the intervention group could be a coincidental finding.

Limitations of the study should be considered. First, data were collected intermittently to reduce participant burden, but this may be associated with a slight inaccuracy in data reporting, and in cost estimates as a consequence [25]. Second, besides monetary investments, participants have to make a time investment, which was not taken into account in the current analysis as this is considered to be captured in the assessment of the quality of life [26]. Furthermore, costs of transportation were not included because the intervention was delivered in participants’ neighbourhoods; therefore distances were short and costs negligible. Third, we included costs and effects during the intervention period up to six months after the end of the intervention. Although the intervention was not cost-saving, beneficial effects on intermediate outcomes were found. Improvements in weight, fasting insulin, dietary intake, and physical activity were observed at 12 months, and most of these improvements were sustained at 18 months [8]. In Li et al.’s review, it was shown that programmes were most cost-effective in the longer term, indicating that short-term studies are limited in their ability to capture the full range of an intervention’s health benefits and cost savings [2]. Therefore, more insight into longer-term cost-effectiveness is needed, and the results of our study should be modelled to a lifetime horizon. We expect more favourable cost-effectiveness on the longer term because diabetes will be postponed or prevented, leading to cost savings in the future.

A strength of the current study is the use of a randomised design in a real-world setting. Furthermore, data were complete for 84% of the measurements. In the event of missing values, multiple imputation techniques were used, which is a status quo method for dealing with missing data [27]. Moreover, we performed the CEA from a societal perspective as recommended by the Dutch guideline for costing analysis in health care [15, 16]. In addition, we performed the evaluation from a health care perspective, the perspective most relevant to health insurance companies which may consider to reimburse the intervention programme.

## Conclusions

In summary, our results indicate that the SLIMMER intervention is more cost-effective from a health care perspective than from a societal perspective. Costs were higher in the intervention group, mostly due to costs of the intervention programme and higher productivity losses. Intervention costs could be decreased to a certain extent to further enhance the cost-effectiveness of the SLIMMER intervention.

## Supplementary information

**Additional file 1.** Detailed description of cost prices [28].

**Additional file 2: Figure S1.** Cost-effectiveness plane from 1000 bootstrap simulation for the SLIMMER intervention compared to usual health care.

## Data Availability

The datasets used and/or analysed during the current study are available from the corresponding author on reasonable request.

## Abbreviations

CEA: Cost-effectiveness analysis
CEAC: Cost-effectiveness acceptability curve
DPP: Diabetes Prevention Program
DPS: Diabetes Prevention Study
GP: General practitioner
ICER: Incremental cost-effectiveness ratio
QALY: Quality adjusted life year
SF-36: Short-form health survey 36
SF-6D: Short-form 6 dimensions
SF-HLQ: Short form health and labour questionnaire
SLIM: Study on Lifestyle intervention and Impaired glucose tolerance Maastricht
SLIMMER: SLIM iMplementation Experience Region Noord- en Oost-Gelderland
WTP: Willingness-to-pay
