# Supplementary material for: Cost-effectiveness of the SLIMMER diabetes prevention intervention in Dutch primary health care: economic evaluation from a randomised controlled trial
Source: BMC Health Serv Res. 2019 Nov 11;19:824. doi: 10.1186/s12913-019-4529-8 (PMC6849241; doi:10.1186/s12913-019-4529-8)
Supplement: Supplementary file 1 — Additional file 1. Detailed description of cost prices [28]. [file 12913_2019_4529_MOESM1_ESM.pdf]

Additional file 1. Detailed description of cost prices

Cost prices of intervention implementers (practice nurse, dietician, and physiotherapist), the project coordinator, and sports clubs' instructors (giving sports clinics) were retrieved from the Dutch guideline for costing analysis in health care [15, 16].

Cost prices of health care utilisation (general practice, dietician, physiotherapist, health care specialist, and hospital days) were retrieved from the Dutch guideline for costing analysis in health care [15, 16]. An overview of exact cost prices can be found in Table 2 and Table A1.

Cost prices of medication were based on summary cost prices for average daily dosages as used in the Netherlands, according to the *Pharmacotherapeutic Compass* [28], augmented by the 3-month delivery tariff charged by pharmacies.

The participant out-of-pocket costs were valued as indicated by the participants themselves.

Productivity losses were assessed with the friction cost approach [15, 16], using a friction period of 115 days. For all participants with a paid job, age- and sex-standardised productivity costs per hour were used, following the Dutch guideline for costing analysis in health care [15, 16].

Table A1. Unit costs for cost categories not mentioned in Table 2

	Unit costs (€)
Direct health care costs	
General practice	
Visit to practice	29.70 per visit
Phone consultation	14.85 per consultation
Home visit	45.61 per visit
Phone contact for medical prescription	14.85 per contact
