# Supplementary material for: Cost-effectiveness of the SLIMMER diabetes prevention intervention in Dutch primary health care: economic evaluation from a randomised controlled trial
Source: BMC Health Serv Res. 2019 Nov 11;19:824. doi: 10.1186/s12913-019-4529-8 (PMC6849241; doi:10.1186/s12913-019-4529-8)

Additional file 2.

Figure A1. Cost-effectiveness plane from 1000 bootstrap simulation for the SLIMMER intervention compared to usual health care

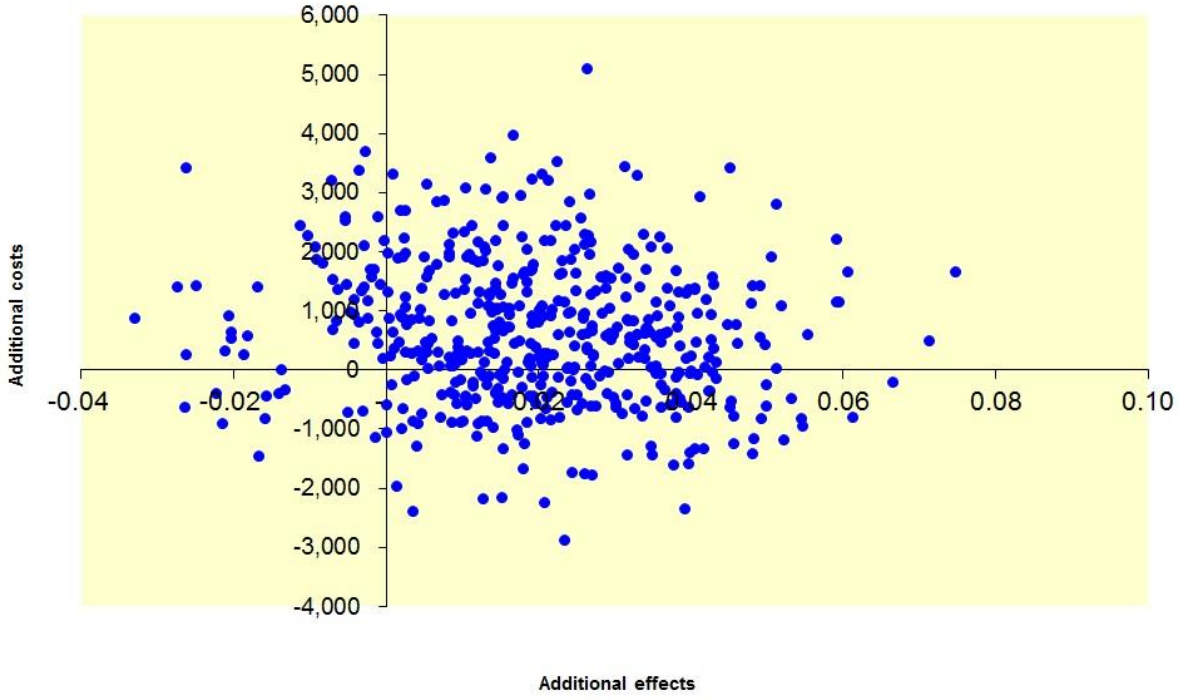

Supplement: Supplementary file 2 — Additional file 2: Figure S1. Cost-effectiveness plane from 1000 bootstrap simulation for the SLIMMER intervention compared to usual health care. [file 12913_2019_4529_MOESM2_ESM.pdf]
